# Activation of intrahepatic CD4^+^CXCR5^+^ T and CD19^+^ B cells is associated with viral clearance in a mouse model of acute hepatitis B virus infection

**DOI:** 10.18632/oncotarget.10688

**Published:** 2016-07-18

**Authors:** Xiao-Fei Song, Ting-Ting Hu, Yu Lei, Hu Li, Li Zhang, Miao Zhang, Bin Liu, Min Chen, Huai-Dong Hu, Hong Ren, Peng Hu

**Affiliations:** ^1^ Key Laboratory of Molecular Biology for Infectious Diseases (Ministry of Education), Institute for Viral Hepatitis, Department of Infectious Diseases, The Second Affiliated Hospital, Chongqing Medical University, Chongqing, China

**Keywords:** acute hepatitis B, hydrodynamic injection, intrahepatic lymphocytes, viral clearance, Immunology and Microbiology Section, Immune response, Immunity

## Abstract

The role of immunity in the pathogenesis of acute hepatitis B virus (HBV) infection is poorly understood. The purpose of this research was to define the intrahepatic immune factors responsible for viral clearance during acute HBV infection. The model of acute HBV infection was established by hydrodynamically transfecting mice with pCDNA3.1-HBV1.3 plasmids which contained a supergenomic HBV1.3-length transgene. The frequency of CD4^+^ CXCR5^+^ T cells, CD19^+^ B cells and their surface molecules in livers, spleens and peripheral blood were detected using flow cytometry. The lymphomononuclear cells isolated from the livers of transfected mice were further stimulated by HBc-derived peptides and then the frequency and cytokine secretion of HBV-specific CD4^+^CXCR5^+^ T cells were detected. We found that the frequency of CXCR5^+^ in CD4^+^ T cells was specifically increased; the expression of PD-1 was decreased while the expression of ICOS was increased on intrahepatic CD4^+^CXCR5^+^ T cells. Although the frequency of CD19^+^ B cells was not affected, the expression of PDL-1, ICOSL and IL-21R on B cells was increased in the livers of mice. The frequency of HBV-specific CD4^+^CXCR5^+^ T cells and the production of IL-21 by intrahepatic CD4^+^CXCR5^+^ T cells of mice with acute HBV infection were increased after stimulation. Furthermore, the expression of function-related molecules of intrahepatic CD4^+^CXCR5^+^ T, including Bcl-6, CXCR5, IL-6, IL-6R, IL-21 and IL-4 in the liver was increased during acute HBV infection. In conclusion, the activation of intrahepatic CD4^+^CXCR5^+^ T cells and B cells was associated with the clearance of HBV during acute infection.

## INTRODUCTION

The hepatitis B virus (HBV) is a kind of hepadnaviridae virus which causes acute and chronic liver disease. However, HBV is not directly cytopathic for the hepatocytes [1, 2] and the immune response is thought to play a critical role in viral clearance and disease pathogenesis during HBV infection. Since HBV acts as a stealth virus early after infection, the early innate immune response does not significantly contribute to the control of viremia during HBV infection. Instead, the specific immune responses play crucial roles in viral clearance and disease pathogenesis during HBV infection [3]. Patients with acute viral hepatitis, who successfully clear the virus, mount a multi-specific polyclonal CD8^+^ and CD4^+^ T cell response to several HBV-encoded antigens [4, 5]. Many studies discuss the immune response in peripheral blood mononuclear cells during HBV infection. However, the full spectrum of intrahepatic immunological factors that involved in the acute HBV infection is not completely defined.

T follicular helper (Tfh) cell is a special subpopulation of CD4^+^ T helper cells that regulate B cell-mediated humoral immune responses. Tfh cells are characterized as high expression of chemokine receptor CXCR5 [6], transcription factor Bcl-6 and the secretion of cytokine IL-21 [7, 8]. CXCR5 allows the migration of Tfh cells and the stable contacting with antigen-primed B cells in B-cell follicles [6]. Tfh cells also provide signals including co-stimulatory molecules CD40L, inducible co-stimulator (ICOS), programmed cell death 1 (PD-1) as well as IL-21 to B cells for their survival, differentiation and proliferation. Recently, Tfh cells have been extensively studied in many infectious diseases including HBV infection. It was reported that circulating CXCR5+CD4+ T cells were expanded in patients with chronic hepatitis B [9–12] and high frequency of circulating CXCR5+CD4+ T cells were associated with HBeAg seroconversion through IL-21 production manner [9]. Our preliminary works have also shown the expansion of circulating Tfh cells and their associated molecules in patients with chronic HBV infection [12]. However, the role of Tfh cells during acute HBV infection was still unknown.

Since the host range of HBV is limited to man and chimpanzees and there was no *in vitro* systems for the propagation of HBV, the experimental approaches to HBV pathogenesis was difficult. To overcome these limitations, the hydrodynamic transfection method was used to establish an HBV infection model [13]. The plasmid which contained an over-length, linear HBV genome was injected intravenously into immunocompetent mice under hydrodynamic conditions to transfect hepatocytes *in vivo* [13, 14]. In this mice model, viral antigens and replicative intermediates were synthesized and virus was secreted into the blood. In the present study, we used a mouse model with hydrodynamic injection of viral DNA for acute HBV infection to investigate the phenotypes and functions of intrahepatic immune cells that participated in viral clearance during acute HBV infection.

## RESULTS

### Construction of a mouse model for acute hepatitis B infection

**Figure 1:**
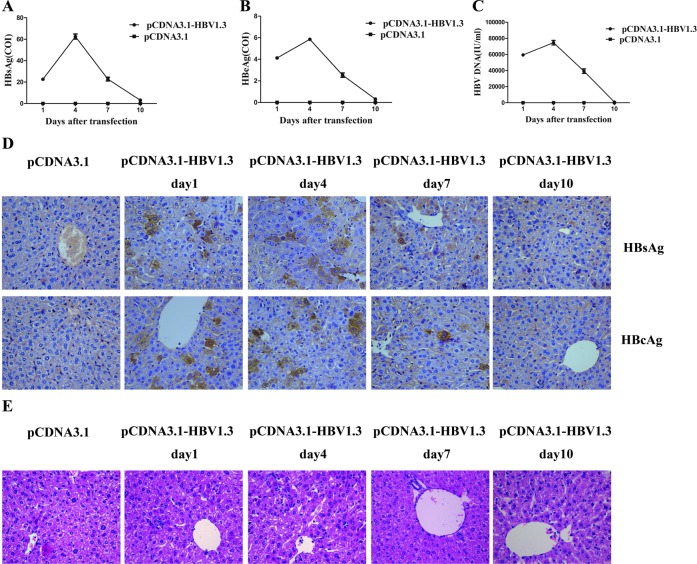
HBsAg, HBeAg and HBV DNA expressed in the serum of transfected mice, and HBsAg and HBcAg expressed in the livers of transfected mice Mice were hydrodynamically transfected with pCDNA3.1-HBV1.3 (*n* = 8 in each time point) or pCDNA3.1 (*n* = 8 in each time point). **A.** The HBsAg level, **(B).** The HBeAg level, **(C)** HBV DNA were determined in mice sera on days 1, 4, 7 and 10 after transfection. Error bars represent the SEM of the data from at least 4 mice per group. **D.** The HBsAg and HBcAg expression was examined using IHC staining of liver tissue sections from mice on days 1, 4, 7 and 10 after transfection. HBsAg+ and HBcAg+ hepatocytes in livers on days 1, 4, 7 and 10 are shown. Original magnification, 40×. **E.** HE stains of liver sections from mice transfected with pCDNA3.1-HBV1.3 or pCDNA3.1 on days 1, 4, 7 and 10. Original magnification, 40×.

To monitor the transfection efficiency, we examined the viremia in the mice days 1, 4, 7 and 10 after hydrodynamic transfection. In the pCDNA3.1-HBV1.3-injection mice, the concentration of HBsAg in the blood serum reached to 22.72 ± 2.44 COI on day 1 and increased to 62.53 ± 4.66 COI on day 4. After this peak, serum HBsAg dropped to 22.79±3.50 COI and 3.13 ± 0.54 COI on days 7 and 10 respectively after transfection (Figure 1A). The concentration of HBeAg in the serum was 4.12 ± 0.29 COI on day 1,which increased to 5.85 ± 0.26 COI at the peak of expression on day 4 and decreased to 2.53 ± 0.41 COI and 0.31 ± 0.04 COI on days 7 and 10, respectively (Figure 1B). HBsAg (Figure 1A) and HBeAg (Figure 1B) were not detected in the control pCDNA3.1 group. As shown in Figure 1C, the average HBV DNA on day 1 was 5.94 ± 0.23 × 10^4^ IU/ml and peaked at 7.46 ± 0.56 × 10^4^ IU/ml on day 4. The HBV DNA subsequently declined on day 7 (3.92 ± 0.57 × 10^4^ IU/ml) and day 10 (0.79 ± 0.09 × 10^3^ IU/ml) in the pCDNA3.1-HBV1.3 group. HBV DNA (Figure 1C) was certainly not detected in the pCDNA3.1 group. Immunohistochemical (IHC) staining of the liver sections also showed that HBsAg+ and HBcAg+ hepatocytes were randomly distributed throughout the liver lobule in the pCDNA3.1-HBV1.3 group (Figure 1D). As control, HBsAg and HBcAg were not detected in the pCDNA3.1 group (Figure 1D). As shown in Figure 1E, necroinflammatory changes were observed in the livers of mice transfected with pCDNA3.1-HBV1.3. These results indicated that viral DNA was successfully transfected into the livers of mice by hydrodynamic injection.

### Increased frequencies of CD4^+^CXCR5^+^ and ICOS^+^CD4^+^CXCR5^+^ T cells, but decreased frequencies of PD-1^+^CD4^+^CXCR5^+^ T cells in the livers of pCDNA3.1-HBV1.3-transfected mice

**Figure 2:**
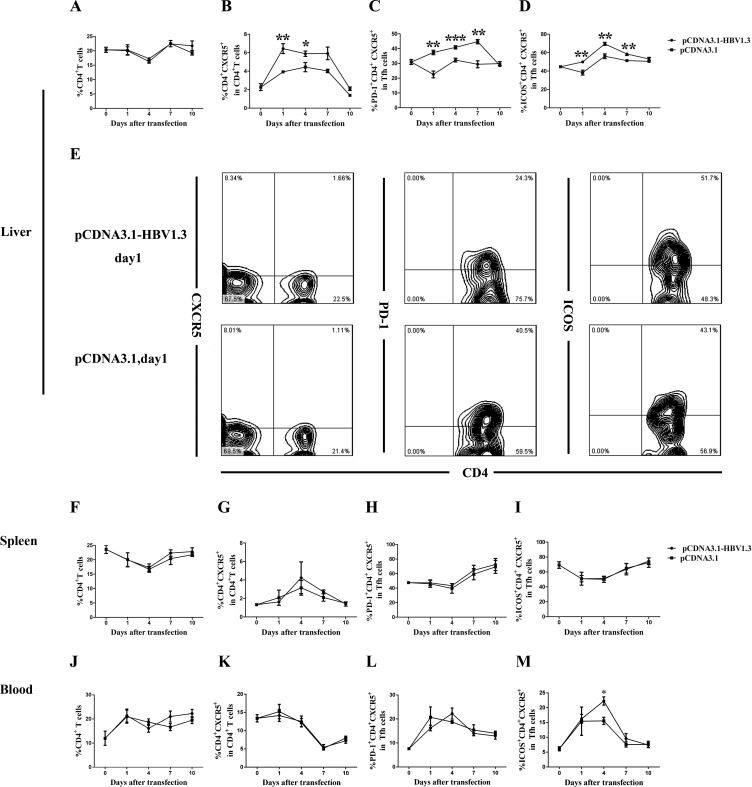
FACS analysis of the frequency of CD4^**+**^T cells, CD4^**+**^CXCR5^**+**^ cells, PD-1^**+**^CD4^**+**^CXCR5^**+**^ and ICOS^**+**^CD4^**+**^CXCR5^**+**^ cells in the livers and spleens of transfected mice Mice were hydrodynamically transfected with pCDNA3.1-HBV1.3 (*n* = 10 in each time point) or pCDNA3.1 (*n* = 10 in each time point). The lymphomononuclear cells in the livers and spleens were isolated and analyzed by flow cytometry. **A.**-**D.** The frequency of CD4^+^T cells, the percentage of CD4^+^CXCR5^+^ cells in CD4^+^ T cells, the percentage of PD-1^+^CD4^+^CXCR5^+^ and ICOS^+^CD4^+^CXCR5^+^ cells in CD4^+^CXCR5^+^ T cells in the livers of mice before transfection or on days 1, 4, 7 and 10 after transfection. **E.** Representative flow chart showing the frequency of intrahepatic CD4^+^ T cells, the percentage of intrahepatic CD4^+^CXCR5^+^ cells in CD4^+^ T cells, the percentage of intrahepatic PD-1^+^CD4^+^CXCR5^+^ and ICOS^+^CD4^+^CXCR5^+^ cells in CD4^+^CXCR5^+^ T cells on day 1 during acute HBV infection. **F.**-**M.** The frequency of CD4^+^ T cells, the percentage of CD4^+^CXCR5^+^ cells in CD4^+^ T cells, the percentage of PD-1^+^CD4^+^CXCR5^+^ and ICOS^+^CD4^+^CXCR5^+^ cells in CD4^+^CXCR5^+^ T cells in the spleens **(F**-**I)** and the peripheral blood **(J**-**M)** of the mice before transfection or on days 1, 4, 7 and 10 after transfection. Error bars represent the SEM of the data. *, ** and *** indicate *p* values of < 0.05, < 0.01 and < 0.001, respectively.

To investigate whether acute HBV infection could affect the phenotype of CD4^+^CXCR5^+^ T cells, the frequency and function-related molecules of CD4^+^CXCR5^+^ T cells in the livers, spleens and peripheral blood of the mice before and after transfection were characterized. The difference of the frequencies of intrahepatic CD4^+^ T cells between two groups was not significant (Figure 2A). However, on days 1 and 4, the frequencies of intrahepatic CD4^+^CXCR5^+^ T cells in CD4^+^ T cells were higher in mice transfected with pCDNA3.1-HBV1.3 than those in mice transfected with pCDNA3.1 (Figure 2B and Figure 2E). Notably, the frequency of PD-1^+^ cells in intrahepatic CD4^+^CXCR5^+^ T cells in the pCDNA3.1-HBV1.3 group were significantly lower than that in the pCDNA3.1 group on days 1, 4 and 7 (Figure 2C and Figure 2E). On the contrary, the frequency of ICOS^+^ cells in intrahepatic CD4^+^CXCR5^+^ T cells was significantly increased on days 1, 4 and 7 in the pCDNA3.1-HBV1.3 group compared with that in the pCDNA3.1 group (Figure 2D and Figure 2E). It is interesting to note that the difference of the frequencies of CD4^+^ T cells, the percentages of CXCR5^+^ cells in CD4^+^ T cells or the percentages of PD-1 ^+^ and ICOS ^+^ cells in CD4^+^CXCR5^+^ T cells in the spleens of the mice between the two groups were not significant (Figure 2F-2I). Furthermore, no significant differences were observed in the frequencies of CD4^+^ T cells, the percentages of CXCR5^+^ cells in CD4^+^ T cells and the percentages of PD-1^+^ cells in CD4^+^CXCR5^+^ T cells in the peripheral blood of the mice between the two groups (Figure 2J - 2L). But on days 4, the percentage of ICOS^+^ cells in CD4^+^CXCR5^+^ T cells was significantly higher in the pCDNA3.1-HBV1.3 group than that in the pCDNA3.1 group (Figure 2M). These results indicated that CD4^+^CXCR5^+^ T cells and their activation were involved in the intrahepatic immune response of viral clearance during acute HBV infection.

### High expression of PDL-1, ICOSL and IL-21R on B cells in the livers of pCDNA3.1-HBV1.3-transfected mice

**Figure 3:**
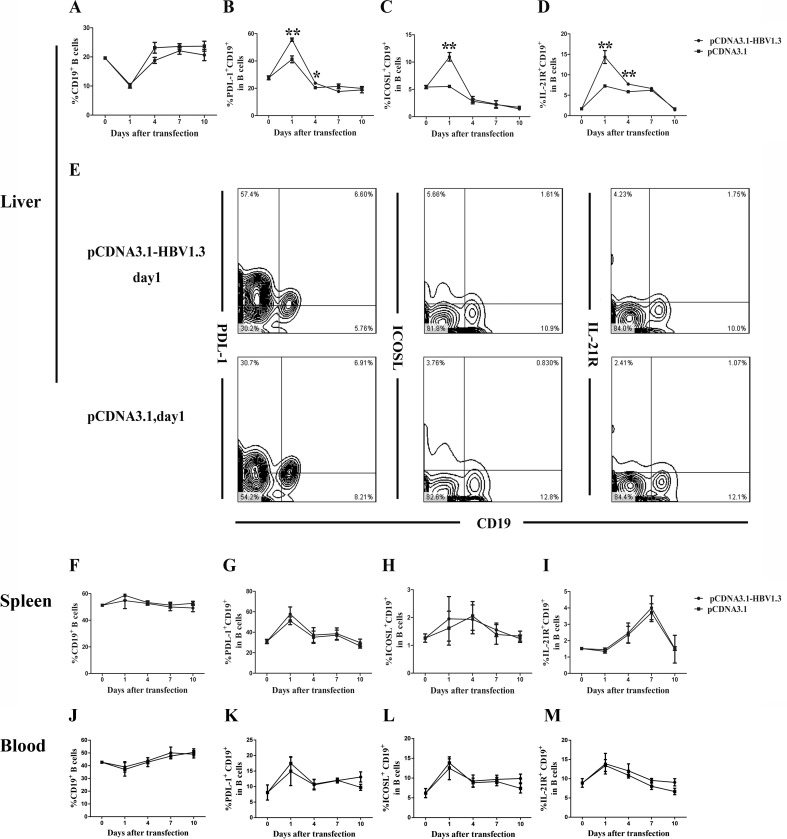
The frequency of CD19^**+**^B cells and the PDL-1, ICOSL and IL-21R expressed on CD19^**+**^ B cells in the livers and spleens of transfected mice Mice were hydrodynamically transfected with pCDNA3.1-HBV1.3 (*n* = 10 in each time point) or pCDNA3.1 (*n* = 10 in each time point). The lymphomononuclear cells in the livers and spleens were isolated and analyzed using flow cytometry. **A.**-**D.** The frequency of CD19^+^ B cells and the PDL-1, ICOSL and IL-21R expressed on CD19^+^ B cells in the livers of mice before transfection or on days 1, 4, 7 and 10 after transfection on days 1, 4, 7 and 10. **E.** Representative flow chart showing the frequency of inthrahepatic CD19^+^ B cells and the PDL-1, ICOSL and IL-21R expressed on inthrahepatic CD19^+^ B cells on day 1 during acute HBV infection. **F.**-**M.** The frequency of CD19^+^ B cells and the PDL-1, ICOSL and IL-21R expressed on CD19^+^ B cells in the spleens **(F**-**I)** and the peripheral blood **(J**-**M)** of mice before transfection or on days 1, 4, 7 and 10 after transfection. Error bars represent the SEM of the data. *and ** indicate *p* values of < 0.05 and < 0.01, respectively.

CD4^+^CXCR5^+^ T cells can promote B cell activation, expansion and differentiation through various means. To assess whether the phenotype of the B cells was affected during acute HBV infection, the frequencies and function-related molecules of B cells, including PDL-1, ICOSL, IL-21R, in B cells from livers, spleens and peripheral blood of the mice were analyzed using flow cytometry. Although no significant difference in the frequency of CD19^+^ B cells was found in the mice livers between the two groups (Figure 3A), the expression of PDL-1 on intrahepatic CD19^+^ B cells were increased on days 1 and 4 in the pCDNA3.1-HBV1.3 group compared with that in the pCDNA3.1 group (Figure 3B and Figure 3E). In addition, the frequency of ICOSL^+^CD19^+^ B cells (Figure 3C and Figure 3E) in the livers of mice transfected with the pCDNA3.1-HBV1.3 was significantly higher than that in the pCDNA3.1 group on day 1. Similarly, the expression of IL-21R in intrahepatic CD19^+^ B cells (Figure 3D and Figure 3E) of mice transfected with the pCDNA3.1-HBV1.3 was also significantly increased on days 1 and 4 than that in the mice transfected with pCDNA3.1. On the contrary, the frequency of CD19^+^ B cells, the expression of PDL-1, ICOSL and IL-21R on CD19^+^ B cells in the spleens and peripheral blood of the mice were not significantly different between the two groups (Figure 3F-3M). The results indicated that the activation of B cells were involved in the intrahepatic immune response of viral clearance during acute HBV infection.

### Increased frequencies of HBV-specific CD4^+^CXCR5^+^ T cells and IL-21^+^CD4^+^CXCR5^+^ T cells in the livers of pCDNA3.1-HBV1.3 transfected mice

**Figure 4:**
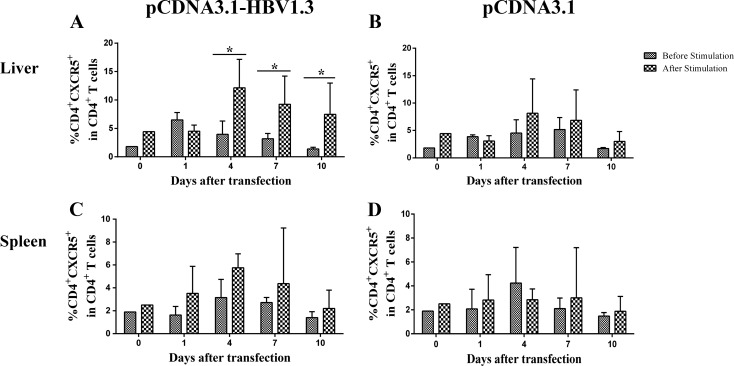
HBV-specific CD4^**+**^ CXCR5^**+**^T cells were detected by flow cytometry through re-stimulating the isolated lymphomononuclear cells from the livers and spleens with HBc-derived peptides HBc1-20 Mice were hydrodynamically transfected with pCDNA3.1-HBV1.3 (*n* = 10 in each time point) or pCDNA3.1 (*n* = 10 in each time point). The lymphomononuclear cells in the livers and spleens were isolated and stimulated by HBc-derived peptides HBc1-20 and analyzed using flow cytometry. **A.**-**B.** The frequency of CD4^+^CXCR5^+^ cells in CD4^+^ T cells in the livers of pCDNA3.1-HBV1.3-transfected mice **(A)** and pCDNA3.1-transfected mice **(B)** before stimulation or after stimulation. C-D. The frequency of CD4^+^CXCR5^+^ cells in CD4^+^ T cells in the spleens of pCDNA3.1-HBV1.3-transfected mice **(C).** and pCDNA3.1-transfected mice **(D)** before stimulation or after stimulation. Error bars represent the SEM of the data. *indicate *p* values of < 0.05.

**Figure 5:**
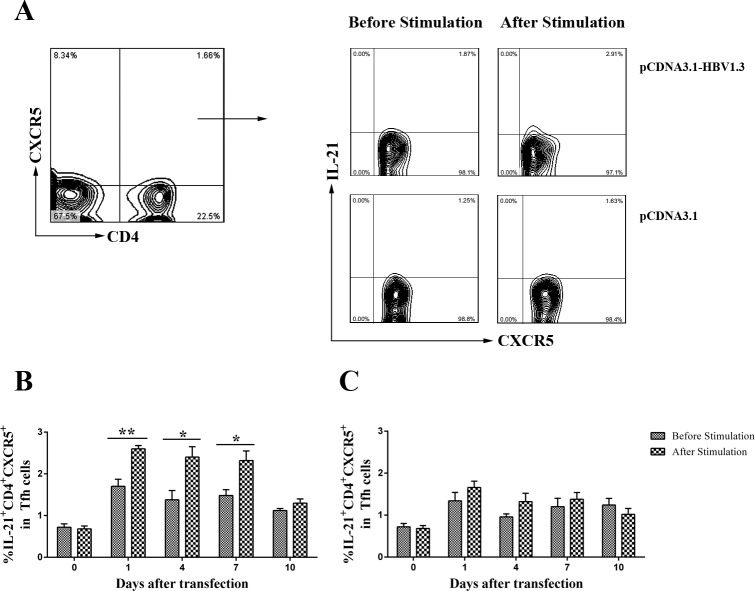
The frequency of IL-21^**+**^CD4^**+**^CXCR5^**+**^T cells in CD4^**+**^CXCR5^**+**^ T cells were detected by flow cytometry before and after re-stimulating the isolated lymphomononuclear cells from the livers with HBc-derived peptides HBc1-20 Mice were hydrodynamically transfected with pCDNA3.1-HBV1.3 (*n* = 10 in each time point) or pCDNA3.1 (*n* = 10 in each time point). The lymphomononuclear cells in the livers were isolated, then the cells were stimulated by HBc-derived peptides HBc1-20 and analyzed using flow cytometry. **A.** Representative flow chart showing the frequency of IL-21^+^CD4^+^CXCR5^+^ T cells in CD4^+^CXCR5^+^ T cells on day 1 in the livers of the transfected mice. **B.** The frequency of IL-21^+^CD4^+^CXCR5^+^ T cells in CD4^+^CXCR5^+^ T cells in the livers of pCDNA3.1-HBV1.3-transfected mice before stimulation or after stimulation. **C.** The frequency of IL-21^+^CD4^+^CXCR5^+^ T cells in CD4^+^CXCR5^+^ T cells in the livers of pCDNA3.1-transfected mice before stimulation or after stimulation. Error bars represent the SEM of the data. *and ** indicate *p* values of < 0.05 and < 0.01, respectively.

In order to examine whether the alteration of CD4^+^ T cells from the pCDNA3.1-HBV1.3-transfected mice was HBV-specific, lymphomononuclear cells from the livers and spleens of the transfected mice were re-stimulating by HBc-derived peptides HBc1-20 and the frequency of CXCR5^+^ in CD4^+^ T cells were analyzed using flow cytometry. Impressively, on days 4, 7 and 10, the frequencies of CXCR5^+^ T cells in CD4^+^ T cells were increased in the livers of pCDNA3.1-HBV1.3-transfected mice after stimulation (Figure 4A). As control, the difference of frequencies of CXCR5^+^ in CD4^+^ T cells from the liver of pCDNA3.1-transfected mice was not significantly before and after stimulation (Figure 4B). However, the frequencies of CXCR5^+^ T cells in CD4^+^ T cells from the spleen were not significantly different before and after stimulation in the two groups (Figure 4C and Figure 4D). Furthermore, the frequency of IL-21 producing CD4^+^CXCR5^+^ T cells was increased in the liver of mice transfected with pCDNA3.1-HBV1.3 group after stimulation by HBV specific peptide (Figure 5A and Figure 5B). This difference was certainly not observed in the livers of pCDNA3.1-transfected mice before and after stimulation (Figure 5C). These results indicated that HBV-specific CD4^+^CXCR5^+^ T cells might be involved in the intrahepatic immune response of viral clearance during acute HBV infection.

### Increased Bcl-6, CXCR5, IL-6, IL-6R, IL-21 and IL-4 mRNA expression in IHLs during acute HBV infection

**Figure 6:**
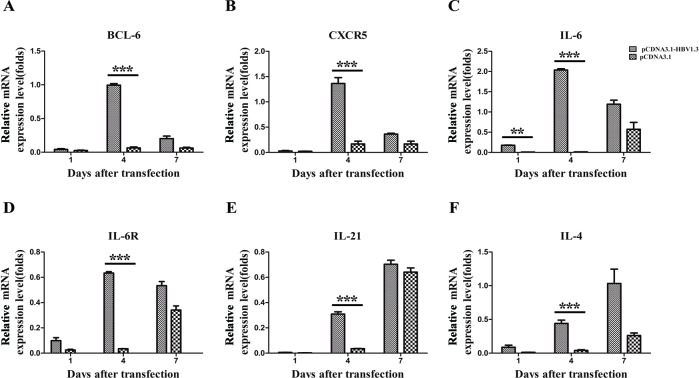
The mRNA expression of BCL-6, CXCR5, IL-6, IL-6R, IL-21, and IL-4 in the livers of transfected mice Mice were hydrodynamically transfected with pCDNA3.1-HBV1.3 (*n* = 5 in each time point) or pCDNA3.1 (*n* = 5 in each time point). **A.**-**F.** The IHLs were isolated by density gradient centrifugation, and total RNA was extracted from IHLs. Bcl-6 mRNA **(A)**, CXCR5 mRNA **(B)**, IL-6 mRNA **(C).**, IL-6R mRNA **(D)**, IL-21 mRNA **(E)** and IL-4 mRNA **(F)** expression in IHLs on days 1, 4 and 7 after transfection. Error bars represent the SEM of the data. **and *** indicate *p* values of < 0.01 and < 0.001, respectively.

Bcl-6 has been verified to be an important transcriptional regulator for the differentiation of Tfh cell [9], while CXCR5 is a conventional marker of Tfh cells. Moreover, a number of cytokines, including IL-6, IL-6R, IL-21 and IL-4, have been implicated in shaping immune responses after viral infection [10–12]. To examine whether the Tfh-related molecules was affected by the HBV infection, the expression of the Bcl-6, CXCR5, IL-6, IL-6R, IL-21 and IL-4 mRNAs in IHLs were analyzed by Real-Time PCR on days 1, 4 and 7 after HBV infection. The mRNA expression of Bcl-6 (Figure 6A), CXCR5 (Figure 6B), IL-6R (Figure 6D), IL-21 (Figure 6E) and IL-4 (Figure 6F) was significantly increased on day 4 in the livers of mice transfected with pCDNA3.1-HBV1.3 compared with that in mice transfected with pCDNA3.1. As shown in Figure 6C, the IL-6 mRNA expression in IHLs was also increased on days 1 and 4 in the pCDNA3.1-HBV1.3 group. These data suggest that Tfh cells and their related cytokines might participate in the intrahepatic immune response for viral clearance after HBV infection.

## DISCUSSION

In this study, we established a mouse model that was hydrodynamically transfected with the pCDNA3.1-HBV1.3 plasmids which contained a 1.3-length transgene HBV for acute HBV infection. We found that the percentage of intrahepatic CXCR5^+^ cells in CD4^+^ T cells was increased during acute HBV infection. PD-1 was down regulated while ICOS was up regulated on CD4^+^CXCR5^+^ T cells in the livers of pCDNA3.1-HBV1.3-transfected mice. The expression of PDL-1, ICOSL and IL-21R on intrahepatic CD19^+^ B cells were significantly increased during acute HBV infection. The frequency and IL-21 producing capacity of HBV-specific CD4^+^CXCR5^+^ T cells was increased in the livers of pCDNA3.1-HBV1.3-transfected mice. We also found that the Bcl-6, CXCR5, IL-6, IL-6R, IL-21 and IL-4 mRNA expression in IHLs was increased during acute HBV infection.

The immune response to viral antigens is thought to be responsible for both liver disease and viral clearance after HBV infection. A series of immune cells are involved in the pathogenesis of HBV infection. Tfh cells have been verified to be crucial for antigen-specific B cell development and the humoral immune response against virus infection [13]. The expansion of Tfh cells has been found in patients with several autoimmune diseases, such as systemic lupus erythematosus [14], rheumatoid arthritis [15] and myasthenia gravis [7]. However, little is known about the role of Tfh cells in the liver of mice during acute HBV infection. Several studies showed that the frequency of CD4^+^CXCR5^+^ T cells was significantly higher in patients with chronic HBV infection than that in healthy subjects [16, 17]. In addition, a previous observation demonstrated that the frequency of intrahepatic CD4^+^CXCR5^+^ T cells in HBV transgenic mice was significantly higher than that in wild-type mice [18]. Our present study revealed that the percentage of intrahepatic CXCR5^+^ cells in CD4^+^ T cells was increased on days 1 and 4 after transfected with pCDNA3.1-HBV1.3. Tfh cells expressed function-related molecules PD-1 and ICOS. PD-1, acting as a potent inhibitory receptor, promotes cognate T-B interactions and provides an inhibitory signal to Tfh cells [19]. On the other hand, ICOS provides key positive signals for the activation, differentiation and effector responses of T cell activation and T cell-dependent B cell responses [20]. We found that the expression of PD-1 on intrahepatic CD4^+^CXCR5^+^ T cells was significantly decreased on days 1, 4 and 7 in the pCDNA3.1-HBV1.3 group. On the contrary, the expression of ICOS on intrahepatic CD4^+^CXCR5^+^ T cells was obviously increased on days 1, 4 and 7 in the pCDNA3.1-HBV1.3 group compared with the pCDNA3.1 group. It was reported that IL-21 plays an important role in B cell proliferation and differentiation [21]. In patients with chronic hepatitis B, circulating Tfh cells benefit hepatitis B e antigen seroconversion through IL-21 [22]. We found that not only the frequency of CXCR5^+^CD4^+^ T cells, but also the frequency of intrahepatic IL-21^+^ producing CD4^+^CXCR5^+^ T cells was increased in the liver of pCDNA3.1-HBV1.3 transfected mice after stimulation with HBV specific antigen. Taken together, our results revealed that acute HBV infection might drive the activation and differentiation of CD4^+^CXCR5^+^ T cells which promote the B cell responses.

Tfh cells interact with B cells through various pairs of molecules, such as PD-1 - PDL-1, ICOS - ICOSL and IL-21 - IL-21R, resulting in the generation of memory B cells and long-lived, antibody-producing plasma cells. A study in mice showed that PD-L1 expression on B cells negatively regulated the expansion of Tfh cell and the expansion and function of Tfh cell were enhanced by treating mice with blocking antibodies to PD-L1 [23]. ICOSL expressed on B cells, which interacted with ICOS expressed on CD4^+^ T cells could induce the expression of Tfh molecules, including IL-21 and Bcl-6 [24]. IL-21R was required to generate Tfh and growth center (GC) B cells, plasma cells and plasmablasts in mice [24]. In our research, the expression of ICOSL, IL-21R on intrahepatic CD19^+^ B cells was higher in the pCDNA3.1-HBV1.3 transfected group than that in the pCDNA3.1 group, suggesting that the ICOSL and IL-21R expressed on intrahepatic B cells might promote the differentiation and function of CD4^+^CXCR5^+^ T cells and cognate CD19^+^ B cells during acute HBV infection. A minor change in the frequency of CD4^+^CXCR5^+^ T cells, CD19^+^ B cells and their surface molecules was also observed in the livers of the pCDNA3.1-transfected mice, suggesting that hydrodynamic injection might induce a minor, intrahepatic inflammatory response during acute HBV infection.

Bcl6 was identified as a transcription factor for Tfh differentiation that induces substantial expression of CXCR5 in naive CD4^+^ T cells. We showed that Bcl-6 and CXCR5 mRNA expression was significantly increased on day 4 in the livers of mice transfected with pCDNA3.1-HBV1.3. These data were in agreement with those of a previous study that found that Bcl6^+^CXCR5^+^ T cells began to appear on day 4 after acute LCMV infection [25]. *In vitro* stimulation of CD4^+^ T cells in the presence of IL-6 could induce Tfh features to some degree [26]. Furthermore, recent studies demonstrated that IL-6 was a key signal for CD4^+^ T cells to initiate Bcl6 induction and Tfh differentiation *in vivo* [27]. Our research showed that the expression of both IL-6 and IL-6R in IHLs was significantly increased in the pCDNA3.1-HBV1.3 transfected group. A previous study in a mouse model of HBV indicated that IL-21 played a vital role in the clearance of HBV antigens [28]. In the present study, the intrahepatic IL-21 expression was also increased in the pCDNA3.1-HBV1.3 transfected group. Our observations suggested that the increased expression of Bcl-6, IL-6, IL-6R, IL-21 and IL-4 in liver might contribute to Tfh cell differentiation and regulate the humoral immune response during acute HBV infection.

In conclusion, our findings revealed that the activation of intrahepatic CD4^+^CXCR5^+^ Tfh cells promoted the cross-talk of CD4^+^CXCR5^+^ T and cognate CD19^+^ B cell during acute HBV infection, which might be associated with the disapperance of virus. Further studies are warranted to examine the specific mechanisms of these immune cells in viral clearance during HBV infection.

## MATERIALS AND METHODS

### Animals

Female, 6-9-week-old C57BL/6J (B6) mice were provided by the Laboratory Animal Center of the Chongqing Medical University. All animals were maintained under specific pathogen-free (SPF) conditions, and the study protocols were approved by the Animal Care Committee guidelines of Chongqing Medical University.

### Hydrodynamic injection of plasmid

Mice were hydrodynamically transfected with the pCDNA3.1-HBV1.3 plasmid which contained a 1.3-length transgene HBV, or a control plasmid pCDNA3.1 (constructed by our laboratory) [27]. Twenty micrograms of plasmids were injected into the tail vein of each mouse in a volume of phosphate buffered saline (PBS) (Double-Helix Biotech) that was equivalent to 8% of the body mass of the mouse. The total volume was delivered within 5-8 seconds [29]. Separate groups of mice were sacrificed on days 1, 4, 7 and 10 after injection. Peripheral blood, livers and spleens were harvested for further analyses.

### Quantitation of viremia

Serum HBsAg, HBeAg and HBV DNA levels were measured in the Clinical Chemistry Laboratory at the Second Affiliated Hospital of Chongqing Medical University. Liver tissues were embedded in paraffin and sectioned. Liver sections were stained with mouse anti-human HBsAg monoclonal antibody (1:100 dilution, MaixinBio) or rabbit anti-human HBcAg polyclonal antibody (1:50 dilution, MaixinBio) following the manufacturer's instructions. Moreover, liver sections were stained with hematoxylin and eosin (HE), and the images were recorded using a light microscopy.

### Isolation of lymphomononuclear cells

For intrahepatic lymphocyte (IHL) isolation, livers were first perfused with 10 ml of PBS *via* portal vein. The livers were digested in RPMI 1640 (Cellgro) containing 0.05% collagenase-IV (Solarbio, Beijing, China) and 0.002% DNase-I (Solarbio, Beijing, China). The IHLs were obtained using density gradient centrifugation. Lymphomononuclear cells in the spleens were isolated by compressing the spleens against the bottom of a Petri dish with the plunger of a syringe. Peripheral blood samples were taken out the plasma and red blood cells were lysed by NH_4_Cl lysis solution.

### Flow cytometry

Lymphomononuclear cells from the livers, spleens and peripheral blood were incubated with fluorochrome-labeled antibodies at 5×10^5^ / tube for 30 min at 4°C, and characterized using a FACS Canto II cytometer (BD Biosciences). For the intracellular cytokine staining, Caltag^TM^, Fix&Perm^®^ reagents (Invitrogen, Carlsbad, CA, USA) were used following the manufacturer's instructions. The following antibodies were used: fluorescein isothiacyanate (FITC)-conjugated anti-CD4, FITC-conjugated anti-CXCR5, FITC-conjugated anti-CD19, Phycoerythrin (PE)-conjugated anti-PD-1, PE-conjugated anti-ICOS, PE-conjugated anti-PDL-1, PE-conjugated anti-ICOSL, PE-conjugated anti-IL-21R, allophycocyanin (APC)-conjugated anti-CD4 (eBioscience, USA), PE-conjugated anti-IL-21 (BD Biosciences, USA) and FITC-, APC- or PE-conjugated isotype antibodies (eBioscience, USA).

Lymphomononuclear cells from the livers and spleens were incubated with HBc-derived peptides HBc1-20 (1 mg / ml)at room temperature for 10 min. HBc-derived peptides HBc1-20 was purchased from Sangon Biotech (Sangon, Shanghai, China). The cells were then washed twice with PBS containing 1% BSA (BD Biosciences), and incubated with anti-CD4, anti-CXCR5 for 30 min at 4°C, and characterized using a FACS Canto II cytometer.

### Real-time PCR

Total RNA was isolated from IHLs using Trizol (Invitrogen, Carlsbad, CA, USA) according to the manufacturer's protocols, and cDNA was synthesized using the PrimeScript RT reagent Kit (TaKaRa, Dalian, China). Real-time PCR was performed on an ABI PRISM 7300 sequence detection system. The cycling parameters were as follows: (I) 1 cycle: 95°C, 30 seconds; and (II) 40 cycles: 95°C, 5 seconds; 60°C, 31 seconds. Primers for BCL-6, CXCR5, IL-6, IL-6R, IL-21, IL-4 and GAPDH were purchased from Sangon Biotech (Sangon, Shanghai, China). The results were normalized to GAPDH mRNA levels and represented using the comparative Ct method.

### Statistical analysis

Data were analyzed using the SPSS version 17.0 software for Windows (SPSS Inc., Chicago, IL.,). Results were expressed as the mean ± standard deviation. Data derived from multiple determinations was subjected to Student's *t-*test and differences were considered statistically significant for *p* values less than 0.05 (two-tailed).

## References

[1] Chisari FV (2000). Rous-Whipple Award Lecture. Viruses, immunity, and cancer: lessons from hepatitis B. The American journal of pathology.

[2] Guidotti LG, Chisari FV (2006). Immunobiology and pathogenesis of viral hepatitis. Annu Rev Pathol Mech Dis.

[3] Yim HJ, Lok ASF (2006). Natural history of chronic hepatitis B virus infection: what we knew in 1981 and what we know in 2005. Hepatology.

[4] Missale G, Redeker A, Person J, Fowler P, Guilhot S, Schlicht H, Ferrari C, Chisari F (1993). HLA-A31-and HLA-Aw68-restricted cytotoxic T cell responses to a single hepatitis B virus nucleocapsid epitope during acute viral hepatitis. The Journal of experimental medicine.

[5] Rehermann B, Fowler P, Sidney J, Person J, Redeker A, Brown M, Moss B, Sette A, Chisari FV (1995). The cytotoxic T lymphocyte response to multiple hepatitis B virus polymerase epitopes during and after acute viral hepatitis. The Journal of experimental medicine.

[6] Schaerli P, Willimann K, Lang AB, Lipp M, Loetscher P, Moser B (2000). CXC chemokine receptor 5 expression defines follicular homing T cells with B cell helper function. The Journal of experimental medicine.

[7] Luo C, Li Y, Liu W, Feng H, Wang H, Huang X, Qiu L, Ouyang J (2013). Expansion of circulating counterparts of follicular helper T cells in patients with myasthenia gravis. Journal of neuroimmunology.

[8] Morita R, Schmitt N, Bentebibel S-E, Ranganathan R, Bourdery L, Zurawski G, Foucat E, Dullaers M, Oh S, Sabzghabaei N (2011). Human Blood CXCR5(+)CD4(+) T Cells Are Counterparts of T Follicular Cells and Contain Specific Subsets that Differentially Support Antibody Secretion. Immunity.

[9] Johnston RJ, Poholek AC, DiToro D, Yusuf I, Eto D, Barnett B, Dent AL, Craft J, Crotty S (2009). Bcl6 and Blimp-1 are reciprocal and antagonistic regulators of T follicular helper cell differentiation. Science.

[10] Dienz O, Eaton SM, Bond JP, Neveu W, Moquin D, Noubade R, Briso EM, Charland C, Leonard WJ, Ciliberto G (2009). The induction of antibody production by IL-6 is indirectly mediated by IL-21 produced by CD4+ T cells. The Journal of experimental medicine.

[11] Yusuf I, Kageyama R, Monticelli L, Johnston RJ, DiToro D, Hansen K, Barnett B, Crotty S (2010). Germinal center T follicular helper cell IL-4 production is dependent on signaling lymphocytic activation molecule receptor (CD150). The Journal of Immunology.

[12] Petrovas C, Yamamoto T, Gerner MY, Boswell KL, Wloka K, Smith EC, Ambrozak DR, Sandler NG, Timmer KJ, Sun X, Pan L, Poholek A, Rao SS, Brenchley JM, Alam SM, Tomaras GD (2012). CD4 T follicular helper cell dynamics during SIV infection. The Journal of clinical investigation.

[13] Sallusto F, Geginat J, Lanzavecchia A (2004). Central memory and effector memory T cell subsets: function, generation, and maintenance. Annu Rev Immunol.

[14] Simpson N, Gatenby PA, Wilson A, Malik S, Fulcher DA, Tangye SG, Manku H, Vyse TJ, Roncador G, Huttley GA (2010). Expansion of circulating T cells resembling follicular helper T cells is a fixed phenotype that identifies a subset of severe systemic lupus erythematosus. Arthritis & Rheumatism.

[15] Wang J, Shan Y, Jiang Z, Feng J, Li C, Ma L, Jiang Y (2013). High frequencies of activated B cells and T follicular helper cells are correlated with disease activity in patients with new-onset rheumatoid arthritis. Clinical and experimental immunology.

[16] Li Y, Ma S, Tang L, Li Y, Wang W, Huang X, Lai Q, Zhang M, Sun J, Li CK (2013). Circulating CXCR5+ CD4+ T cells benefit hbeag seroconversion through IL-21 in patients with chronic HBV infection. Hepatology.

[17] Xing T, Xu H, Yu W (2013). Role of T follicular helper cells and their associated molecules in the pathogenesis of chronic hepatitis B virus infection. Experimental and therapeutic medicine.

[18] Feng J, Lu L, Hua C, Qin L, Zhao P, Wang J, Wang Y, Li W, Shi X, Jiang Y (2011). High frequency of CD4+ CXCR5+ TFH cells in patients with immune-active chronic hepatitis B. PloS one.

[19] Dorfman DM, Brown JA, Shahsafaei A, Freeman GJ (2006). Programmed death-1 (PD-1) is a marker of germinal center-associated T cells and angioimmunoblastic T-cell lymphoma. The American journal of surgical pathology.

[20] Tafuri A, Shahinian A, Bladt F, Yoshinaga SK, Jordana M, Wakeham A, Boucher L-M, Bouchard D, Chan VS, Duncan G (2001). ICOS is essential for effective T-helper-cell responses. Nature.

[21] Konforte D, Simard N, Paige CJ (2009). IL-21: an executor of B cell fate. Journal of immunology.

[22] Li Y, Ma S, Tang L, Li Y, Wang W, Huang X, Lai Q, Zhang M, Sun J, Li CK, Abbott WG, Naoumov NV, Zhang Y, Hou J (2013). Circulating chemokine (C-X-C Motif) receptor 5(+) CD4(+) T cells benefit hepatitis B e antigen seroconversion through IL-21 in patients with chronic hepatitis B virus infection. Hepatology (Baltimore, Md).

[23] Hams E, McCarron MJ, Amu S, Yagita H, Azuma M, Chen L, Fallon PG (2011). Blockade of B7-H1 (programmed death ligand 1) enhances humoral immunity by positively regulating the generation of T follicular helper cells. The Journal of Immunology.

[24] Choi YS, Kageyama R, Eto D, Escobar TC, Johnston RJ, Monticelli L, Lao C, Crotty S (2011). ICOS receptor instructs T follicular helper cell *versus* effector cell differentiation *via* induction of the transcriptional repressor Bcl6. Immunity.

[25] Choi YS, Yang JA, Crotty S (2013). Dynamic regulation of Bcl6 in follicular helper CD4 T (Tfh) cells. Current opinion in immunology.

[26] Nurieva RI, Chung Y, Martinez GJ, Yang XO, Tanaka S, Matskevitch TD, Wang Y-H, Dong C (2009). Bcl6 mediates the development of T follicular helper cells. Science.

[27] Choi YS, Eto D, Yang JA, Lao C, Crotty S (2013). Cutting Edge: STAT1 Is Required for IL-6-Mediated Bcl6 Induction for Early Follicular Helper Cell Differentiation. The Journal of Immunology.

[28] Publicover J, Goodsell A, Nishimura S, Vilarinho S, Wang ZE, Avanesyan L, Spolski R, Leonard WJ, Cooper S, Baron JL (2011). IL-21 is pivotal in determining age-dependent effectiveness of immune responses in a mouse model of human hepatitis B. The Journal of clinical investigation.

[29] Yang PL, Althage A, Chung J, Chisari FV (2002). Hydrodynamic injection of viral DNA: a mouse model of acute hepatitis B virus infection. Proceedings of the National Academy of Sciences.

